# Application of a new ultrasound criterion for the diagnosis of polycystic ovary syndrome

**DOI:** 10.3389/fendo.2022.915245

**Published:** 2022-09-02

**Authors:** Iván Giménez-Peralta, Mariela Lilue, Nicolás Mendoza, Jan Tesarik, Marina Mazheika

**Affiliations:** ^1^ Unidad de Reproducción Hospital Mediterráneo, Almería, Spain; ^2^ Instituto Palacios, Madrid, Spain; ^3^ Department of Obstetrics and Gynecology, University of Granada, Granada, Spain; ^4^ Clínica MAR&Gen, Granada, Spain

**Keywords:** pcos, ultrasound follicular count, hyperandrogenism, insulin resistance, infertility

## Abstract

**Objective:**

To define which ultrasound criteria could replace the classic Rotterdam criteria as the best indicator of the risk of developing endocrine–metabolic changes in women with polycystic ovary syndrome (PCOS).

**Materials and methods:**

This multicenter cross-sectional study included 200 women with PCOS and one control group of 111 women without PCOS. The primary outcomes to be considered were follicular count, hirsutism, total testosterone levels, free androgen index (FAI), and insulin sensitivity (HOMA-IR), and the secondary outcome was the anti-Müllerian hormone (AMH) level.

**Results:**

The main finding in this study points toward a different ultrasound criterion—23 or more follicles of any size in at least one ovary, which is postulated as an alternative to the classic criterion described in the Rotterdam consensus. This criterion correlates better with the other two PCOS criteria and also identifies women at increased risk of hirsutism (Ferriman–Gallwey score: 6.08 ± 3.54 vs. 4.44 ± 3.75, *p* < 0.0001), total testosterone levels (2.24 ± 0.298 vs. 1.42 ± 1.530, *p* = 0.0001), FAI (4.85 ± 0.83 vs. 2.12 ± 1.93, *p* < 0.001), and insulin resistance (HOMA-IR: 1.74 ± 0.182 vs. 1.504 ± 0.230, *p* = 0.001) more accurately. Regarding AMH, large differences in their mean values were observed between the groups (7.07 vs. 4.846 ng/ml, *p* = 0.000). However, these differences depended on age.

**Conclusion:**

The ovarian ultrasound examination with 23 or more follicles of any size in any of the ovaries constitutes a powerful tool to accurately diagnose PCOS and to associate it with metabolic–endocrine processes such as hyperandrogenism and insulin resistance.

## Introduction

Polycystic ovary syndrome (PCOS) is a complex and heterogeneous disease that involves menstrual dysfunction and compromised fertility, as well as metabolic disorders. The diagnosis of PCOS was reached in 2003 by a consensus in Rotterdam by the European Society of Human Reproduction and Embryology and the American Society for Reproductive Medicine, and these are the most broadly used criteria (1) requiring two out of the following three criteria: clinical and/or biochemical hyperandrogenism (HA), oligo- or anovulation, and polycystic ovary morphology (PCOM) on ultrasound (2).

However, the current diagnostic criteria for PCOS (the Rotterdam criteria) overstate its prevalence, which on many occasions hinders a correct clinical–prognostic correlation (2, 3). The most discussed criterion was the ultrasonographic appearance, although other criteria that could replace the current ultrasound criterion have also been proposed, such as the determination of the levels of the anti-Müllerian hormone (AMH).

The classical polycystic appearance is presumed to be due to disordered folliculogenesis, which increases the ratio of growing follicles to resting follicles (4), probably due to the effect of an excess of androgens on early follicular growth (5). However, PCOM may be a normal finding in normal ovulatory women with a normal hormonal and biochemical profile.

The primary objective of the study was to define which ultrasound criteria could replace the classic one as the best indicator of the risk of developing endocrine–metabolic changes in patients with PCOS. The secondary objective was to determine if a high level of AMH could be used to discriminate women diagnosed with PCOS with more severe endocrine and/or metabolic disorders.

## Materials and methods

### Study design

A multicenter cross-sectional study was conducted at three gynecology units in Spain from February 2017 to February 2020: Clinica MAR&Gen in Granada, Preventive Medicine and Public Hospital General Universitario Ciudad Real, and Unidad de Reproducción Mediterráneo in Almería. The study was performed in accordance with the Declaration of Helsinki and good clinical practice guidelines.

### Subjects

The inclusion criteria include women aged between 18 and 40 years with PCOS according to the Rotterdam criteria. Additionally, a group of 111 women in the same age range without PCOS who went to the same centers during the time of the study were also recruited to build a control group.

The exclusion criteria were as follows:

* pregnant women
* women with endocrine or other pathologies that cause cycle disorders or HA
* women who have taken pharmacological treatment that affects the menstrual cycle or biochemical profile related to the study

### Objectives

The primary objectives to be considered were follicular count, hirsutism, testosterone levels, and insulin resistance, and the secondary outcome was the AMH level.

### Methods

All the participants attended spontaneously for some gynecological concern, not necessarily related to PCOS, and were offered the chance to be included in the study or reject it freely. They underwent a complete medical history check. At screening, visits were standardized across all study sites, and each site used identical case report forms, including toxic habits, physical activity and exercise, and clinical history. Body mass index (BMI) was calculated. Hirsutism was assessed in all participants using the Ferriman–Gallwey hirsutism scoring scale. The free androgen index (FAI) was calculated from total testosterone and SHBG using the total T equation (nmol/L) × 100/SHBG (nmol/L), with a normal value <4–4.5. We used the homeostasis model assessment of insulin resistance (HOMA-IR) as a measure of insulin resistance (IR). Insulin levels were determined by a chemiluminescence immunoassay, and the total plasma testosterone concentrations were determined using enzyme-linked fluorescence assay (ELFA).

All ultrasound examinations were carried out with high-resolution transvaginal ultrasonography (Aplio 500 and 8 MHz multifrequency transducers). Each center had one single operator who did all the examinations, and the three operators received the same training in ultrasound. The preset used was the one in which each operator felt most comfortable, in order to obtain the best possible imaging. The follicle count was determined manually without a specific software. For this purpose, sweeping in planes through each ovary was the chosen technique.

Ultrasound examinations were conducted in the early follicular phase of the menstrual cycle or when no dominant follicles or active corpus luteum was present. The operators searched for follicles of any size excluding those which showed luteinization or those whose largest diameter was above 18 mm. Follicle number per ovary (FNPO) and ovarian volume (OV) were assessed throughout each ovary. OV was determined in the largest cross-section of each ovary. If a cystic structure was detected (e.g., corpus luteum or unspecified cyst), then OV measurement was excluded for both ovaries. Mean values between ovaries were calculated and rounded to the nearest whole numbers. Thereafter, we used these criteria to divide women with PCOS into two groups—the experimental group (EG): 82 PCOS women with 23 or more FNPO in one of the ovaries and the control group (CG): 118 PCOS women with 12–22 FNPO in one of the ovaries. The normal group (NG) consisted of 111 women who would not fulfill any criteria for PCOS.

### IRB ethical approval

The study was approved by the Committee of Ethics and Biomedical Investigation of Andalucia, Spain (“Portal de Etica e Investigacion Biomedica de Andalucia”). Patients signed an informed consent document informing them about the procedure; prior to this, the procedure was explained to them, making sure they understood it. All of the patients from the three groups signed an informed consent document informing them about the procedure, and all of them claimed to understand it despite their social status.

### Statistical methodology

To calculate the number of follicles that best predicts the diagnosis of PCOS, an analysis of the diagnostic performance curve (ROC curve) was performed, calculating sensitivity and specificity.

Frequencies, percentages, and chi-square tests were performed on qualitative variables. For quantitative variables, the mean, standard deviation (SD), and 95% confidence intervals were obtained, and the asymptotic *t*-test or bootstrap technique for the *t*-test was performed to compare the groups.

Additionally, statistical multivariate modeling was applied to check differences between groups regarding the evolution of parameters, which used multivariate linear mixed regression models and intrasubject random effect and was fitted with the patients’ characteristics.

To evaluate the effect of age on the risk of HA or IR, we grouped the participants into three age groups (18–25, 26–35, and over 35 years) and then calculated the raw OR and the OR for each age stratum/group. By proceeding this way, we estimated the risk of each age group. A Woolf homogeneity test was performed to evaluate the difference between the specific odds ratios of each stratum and their mean. An OR estimator adjusted for the age variable was combined and calculated. Statistical data were obtained through the program R Project 3.3.

## Results

**Figure 1:**
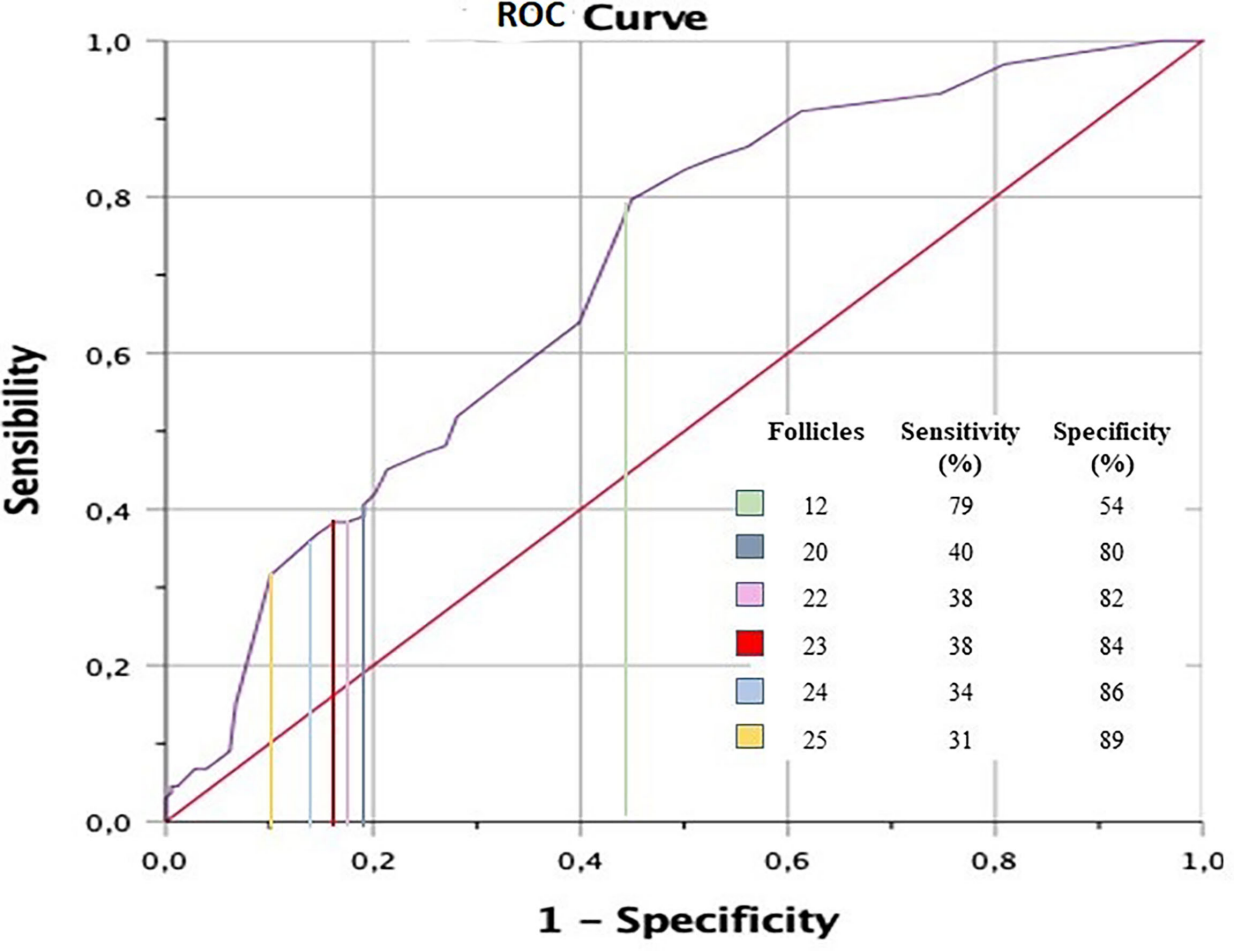
Area under the curve analysis.

The study included 200 PCOS women. First, the analysis of the area under the ROC curve (see **Figure 1**) showed that the count of 23 or more FNPO of any size in any of the ovaries was the value with the best coefficient of sensitivity and specificity to diagnose PCOS.

The BMI, educational level, and physical exercise of the women who participated in the study are shown in **Table 1**. The table shows significant differences between women with or without PCOS, but not between the two groups of women with PCOS.

**Table 1:** BMI, physical activity, and educational level of the different groups.

		Study group	Control group	Normal group	*p*-value
Age (years)		26.56 ± 0.772	28.44 ± 0.51	31.6 ± 0.59	<0.0001
BMI (kg/m^2^)		24.00 ± 0.67	23.3 ± 0.43	22.9 ± 0.55	0.189
Physical activity	None	63.8%	55.6%	37.5%	0.183
	Some	36.2%	44.4%	62.5%	
Education level	No qualifications	2.6%%	1.9%	0%	0.923
	Primary or secondary studies	71.1%	72.2%%	71.4%%	
	Medium level or university studies	26.3%	25.9%	28.6%	

BMI, body mass index.

Regarding the total testosterone level, significant differences were observed between the EG and the other two groups, as well as between the CG and the NG (**Table 2**). Significant differences in the percentage of clinical HA have also been observed between the EG and the other two groups, as well as between the CG and the NG (**Table 2**).

**Table 2:** Hormone and clinical data of the different groups.

		Study group	Control group	Normal group	*p*-value
Total testosterone (nmol/L)		2.24 ± 0.298	1.42 ± 1.530	1.11 ± 0.201	0.0001
FAI		4.85 ± 0.83	2.12 ± 1.93	0.77 ± 1.24	<0.0001
Ferriman–Gallwey		6.08 ± 3.54	4.44 ± 3.75	2.21 ± 2.66	<0.0001
Signs of HA	Yes	41.5%	24.6%	3.6%	<0.0001
	No	58.5%	75.4%	96.4%	
Insulin resistance	yes	15.9%	6.8%	3.6%	0.0094
	No	84.1%	93.2%	96.4%	
Insulin (µUI/ml)		8.24 ± 0.888	6.28 ± 0.990	5.54 ± 0.416	0.0006
HOMA-IR index		1.74 ± 0.182	1.504 ± 0.230	1.15 ± 0.952	0.001
AMH (ng/ml)		7.07 ± 3.94	4.85 ± 2.34	2.49 ± 2	<0.0001

AMH, anti-Müllerian hormone; FAI, free androgen index; HA, hyperandrogenism; HOMA, homeostasis medical assessment.

Regarding HOMA-IR and insulin, differences were observed between the NG and the other groups, but no differences appeared between the CG and the NG. The percentage of women with IR was also higher in the EG compared to the other groups, but not between the CG and the NG (**Table 2**).

Age is inversely related to the number of follicles, but in PCOS women involved in our study, this is not a modifying factor of the effect: when calculating the OR for each of the variables, stratifying it by the established age groups, the adjusted OR and the raw OR of these variables differ very little (Woolf homogeneity test).

Regarding the determination of AMH, large differences in their mean values were observed between groups (NG = 2.495 ng/ml; CG = 4.846 ng/ml; EG = 7.07 ng/ml, *p* = 0.000). However, these differences depended on age (OR = 0.33 for those under the age of 26, 0.64 for those between 26 and 35, and 0.74 for those over 35): the higher the age, the greater the difference between groups.

## Discussion

The main finding of this study points toward a different ultrasound criterion: 23 or more follicles of any size in at least one ovary, which is postulated as an alternative to the classic criterion described in the Rotterdam consensus (more than 12 follicles in at least one ovary of less than 10 mm in volume) (1). This criterion correlates better with the other two PCOS criteria and also identifies women at increased risk of IR and HA more accurately.

**Figure 2:**
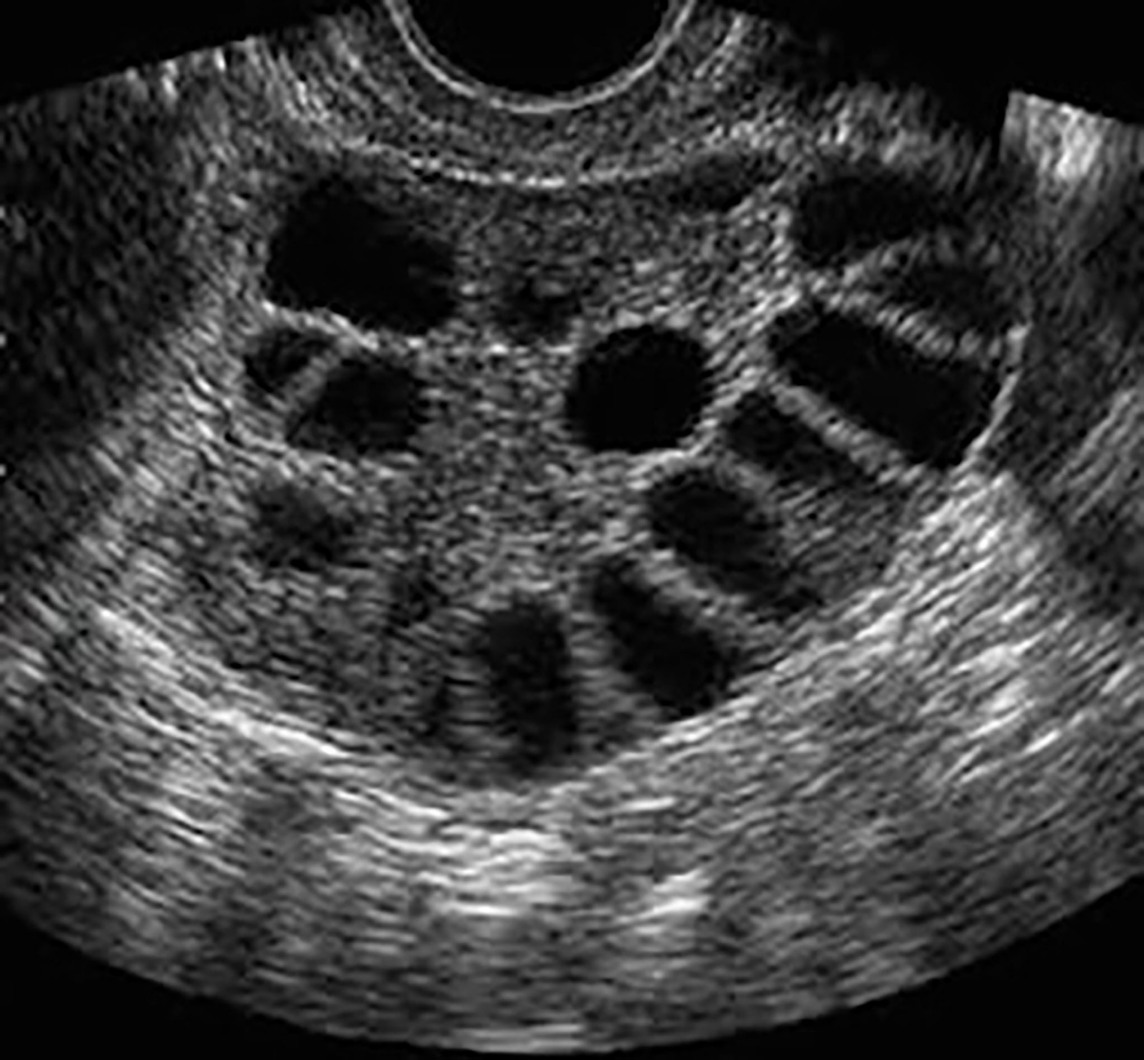
Typical appearance of an ovary with polycystic morphology.

To our knowledge, this is the first comparative study to be performed on women with PCOS using this criterion. Although there are discrepancies in the acceptance of the ultrasound criteria agreed in Rotterdam, currently, there is no international consensus to modify them, except for adolescents and young women, in whom it has been proposed to extend the follicular count to 20 (6–8). **Figure 2** shows the typical appearance of a polycystic ovary on 2D ultrasound.

A comprehensive study carried out by Teede and collaborators suggests that the evidence upon which many study and diagnostic criteria are based is weak, and therefore, the accuracy for such purpose must be refined (9).

This study was proposed with the aim of seeking a new ultrasound criterion, which could avoid overdiagnosis and better define its endocrine and metabolic prognoses. Indeed, numerous studies have shown that the sole presence of PCOM according to the criteria commonly used (nowadays, the Rotterdam criteria are the most commonly accepted, which includes the presence of 12 follicles or more in at least one ovary, and these follicles must measure below 9 mm in diameter) does not predispose to PCOS in women with regular menses; as a matter of fact, PCOS can be ruled out in approximately half of the women with PCOM (10).

PCOS is a heterogeneous condition and its management depends on the reason for consultation and who the specialist who cares for these women is. In this sense, too much importance is given to the ultrasound image when women are treated in gynecology consultations.

Our volunteers come from gynecology consultations, the most common place to deal with PCOS. Consequently, the selection bias that *a priori* could weaken our study does not seem to be so relevant. Another limitation is that a prospective analysis of possible endocrine–metabolic events associated with PCOS cannot be made due to the cross-sectional design of our study.

The analysis of the area under the ROC curve showed that 23 or more follicles of any size in any of the ovaries were the best predictor to diagnose PCOS. With this value, we evaluated the raw OR for each independent variable and then the adjusted OR to present HA according to the Ferriman–Gallwey scale. Concerning FAI and IR, we found an increased risk of more than 100% for each variable when there were more than 22 follicles: specifically, 119% to have HA, 238% to have an elevated FAI, and 168% to have IR (**Table 3**). Women with PCOS included in the EG also showed higher levels of total testosterone and insulin.

**Table 3:** Odds ratio of acquiring HA or IR or having an elevated FAI when 22 or more follicles are observed.

	OR	OR_a_ = exp(lnOR^w^)	IC_95%_
Hyperandrogenism (Ferriman–Gallwey)	2.19	1.80	1.65–3.21*p* < 0.001
HOMA-IR	2.68	2.37	1.66–3.29*p* < 0.001
FAI	3.38	3.09	1.67–4.09*p* < 0.001

FAI, free androgen index; HOMA-IR, homeostasis model assessment of insulin resistance; OR, raw OR; OR_a_, adjusted OR.

On the other hand, no differences in insulin levels or HOMA-IR index were observed between women with PCOS with less than 23 follicles (CG) and the NG. It is interesting to highlight in previous studies that because of the higher prevalence of endocrine–metabolic processes in patients with PCOS, metabolic disorders are not included in their definition, since the existence of a direct relationship between them is unknown. However, in the current study, it has been observed that the value of HOMA-IR is related to a greater number of follicles and that the ultrasound criteria that we propose can redefine PCOS women at risk of IR or diabetes mellitus in adults.

Different studies have compared various methodologies used and FNPO showed to be superior (11–13). Regarding comparison to biochemical markers such as AMH, FNPO is superior in women under 30, but not above that age when the cutoff limit is 12 (14, 15). Also, the 2D transvaginal route has shown to be the best route available (16, 17).

Regarding the value of AMH, an extensive bibliography has suggested it as a possible alternative to ultrasound due to the direct relationship between its plasma values and the count of antral follicles (18, 19). However, its use has not been extended due to the fact that there is great variability observed in its levels, explained by the great clinical heterogeneity of the pathology as well as in the analytical determination method used, and the value above which to diagnose PCOS is not defined either (20). In our study, the determination of the AMH shows that its values are also different between the two groups, being higher in women with the new ultrasound criterion, and although influenced by age, this criterion does not seem appropriate to point to women with PCOS with increased metabolic or endocrine risk.

## Conclusion

In conclusion, PCOS is a prevalent condition which may be overdiagnosed with the current diagnostic criteria. The ultrasound image with 23 or more FNPO in any of the ovaries constitutes a powerful tool to accurately diagnose PCOS and to associate it with high risks for developing metabolic–endocrine processes such as HA and IR. This criterion could avoid overdiagnosis of this syndrome and identify more accurately those women at higher risk of metabolic or biochemical disorders due to PCOS.

## Data availability statement

The raw data supporting the conclusions of this article will be made available by the authors, without undue reservation.

## Ethics statement

The studies involving human participants were reviewed and approved by Universitybof Granada Ethics Committee. The patients/participants provided their written informed consent to participate in this study.

## Author contributions

All authors listed have made a substantial, direct, and intellectual contribution to the work, and approved it for publication.

## Acknowledgments

The statistical analyses have been made by Ramon Ferri, Department of Statistics, University of Granada. Your English Lab has edited the English language in this text. This study is part of the doctoral thesis of IG.

## Conflict of interest

The authors declare that the research was conducted in the absence of any commercial or financial relationships that could be construed as a potential conflict of interest.

## Publisher’s note

All claims expressed in this article are solely those of the authors and do not necessarily represent those of their affiliated organizations, or those of the publisher, the editors and the reviewers. Any product that may be evaluated in this article, or claim that may be made by its manufacturer, is not guaranteed or endorsed by the publisher.
